# Fully automated radiolabeling of [^68^Ga]Ga-EMP100 targeting c-MET for PET-CT clinical imaging

**DOI:** 10.1186/s41181-023-00213-3

**Published:** 2023-10-16

**Authors:** Timofei Rusu, Matthieu Delion, Charlotte Pirot, Amaury Blin, Anita Rodenas, Jean-Noël Talbot, Nicolas Veran, Christophe Portal, Françoise Montravers, Jacques Cadranel, Aurélie Prignon

**Affiliations:** 1 THERANOSCAN Clinical Research Group Sorbonne University, Tenon Hospital AP-HP, Paris, France; 2 Positron Molecular Imaging Laboratory (LIMP) UMS28 Small Animal Phenotyping, Sorbonne University, Paris, France; 3 Nuclear Medicine Imaging Department and Radiopharmacy, Tenon Hospital AP-HP, Paris, France; 4 Institut National des Sciences et Techniques Nucléaires (INSTN), Saclay, France; 5 CHRU de Nancy Pôle Pharmacie : Centre Hospitalier Régional Universitaire de Nancy Pôle Pharmacie, Nancy, France; 6 Edinburgh Molecular Imaging, Edinburgh, UK; 7 Service de Pneumologie et Oncologie Thoracique, APHP – Hôpital Tenon and Sorbonne Université, Paris, France; 8 Radiopharmacist – Hôpital Tenon Assistance Publique – Hôpitaux de Paris, Paris, France

**Keywords:** [^68^Ga]Ga-radiopharmaceuticals, [^68^Ga]Ga-EMP100, c-MET, PET imaging

## Abstract

**Background:**

c-MET is a transmembrane receptor involved in many biological processes and contributes to cell proliferation and migration during cancer invasion process. Its expression is measured by immunehistochemistry on tissue biopsy in clinic, although this technique has its limitations. PET-CT could allow in vivo mapping of lesions expressing c-MET, providing whole-body detection. A number of radiopharmaceuticals are under development for this purpose but are not yet in routine clinical use. EMP100 is a cyclic oligopeptide bound to a DOTA chelator, with nanomolar affinity for c-MET. The aim of this project was to develop an automated method for radiolabelling the radiopharmaceutical [^68^Ga]Ga-EMP100.

**Results:**

The main results showed an optimal pH range between 3.25 and 3.75 for the complexation reaction and a stabilisation of the temperature at 90 °C, resulting in an almost complete incorporation of gallium-68 after 10 min of heating. In these experiments, 90 µg of EMP-100 peptide were initially used and then lower amounts (30, 50, 75 µg) were explored to determine the minimum required for sufficient synthesis yield. Radiolysis impurities were identified by radio-HPLC and ascorbic acid and ethanol were used to improve the purity of the compound. Three batches of [^68^Ga]Ga-EMP100 were then prepared according to the optimised parameters and all met the established specifications. Finally, the stability of [^68^Ga]Ga-EMP100 was assessed at room temperature over 3 h with satisfactory results in terms of appearance, pH, radiochemical purity and sterility.

**Conclusions:**

For the automated synthesis of [^68^Ga]Ga-EMP100, the parameters of pH, temperature, precursor peptide content and the use of adjuvants for impurity management were efficiently optimised, resulting in the production of three compliant and stable batches according to the principles of good manufacturing practice. [^68^Ga]Ga-EMP100 was successfully synthesised and is now available for clinical development in PET-CT imaging.

## Background

c-MET is a transmembrane receptor with tyrosine kinase activity that is activated by its physiological ligand, hepatocyte growth factor. This receptor plays a key role in many physiological processes (embryogenesis, wound healing, etc.). When involved in cancer biology, it activates several intracellular signaling pathways leading to increased proliferation, migration and metastasis of cancer cells through the epithelial-mesenchymal transition process (Sung et al. 2016). This aberrant signalling is found in many primary cancers, including kidney, colorectal and non-small cell lung cancer (NSCLC) (Baldacci et al. 2018; Duplaquet et al. 2018; Gherardi et al. 2012; Ma et al. 2008; Salgia 2017; Zhang et al. 2018).

Currently, in routine clinical practice, patient eligibility for targeted MET therapy is determined on tissue biopsy by immunohistochemistry (IHC) using the specific antibody SP44 (Spigel et al. 2014), by fluorescent in situ hybridisation (FISH) or by next-generation sequencing (NGS) of the *MET* gene. However, these techniques, especially IHC, have limitations as they do not reflect the variability of c-MET expression over time, nor the heterogeneity within a tumour lesion or between different tumour sites. In addition, they rely on the availability of tissue samples, which is not always the case, especially for locations that are inaccessible for biopsy.

**Fig. 1:**
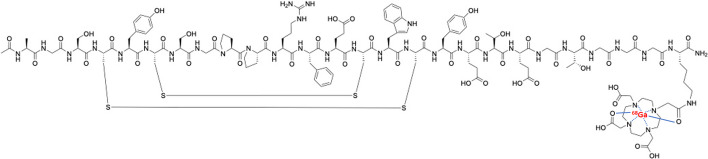
[^68^Ga]Ga-EMP100

Macroscopic PET-CT nuclear imaging has the potential to map c-MET-expressing lesions throughout the body overcoming most of the limitations of the conventional patho-molecular techniques used. Moreover, PET-CT molecular imaging provides non-invasive, real-time detection with high sensitivity and specificity, and allows quantitative analysis of binding intensity using standardized uptake value (SUV) measurement or derived quantification methods. To date, a number of radiopharmaceuticals (RPs) targeting the MET pathway, such as antibodies, peptides or small molecules, have been radiolabelled to detect the sites of various cancers ([^64^Cu]Cu-NOTA-rh-HGF, [^89^Zr]Zr-onartuzumab, [^18^F]F-AH113804, [^11^C]C-SU11274) (Luo et al. 2015; Jagoda et al. 2012; Arulappu et al. 2016; Wu et al. 2010), but there is currently no routine clinical use for them. Targeted therapies for the MET pathway are based mainly on tyrosine kinase inhibitors such as (crizotinib, capmatinib and tepotinib) (Remon et al. 2023), the MET-specific monoclonal antibody onartuzumab (Spigel et al. 2017) or the drug conjugated antibody ABBV-399 (Wang et al. 2017). However, a specific c-MET radioligand in PET could open the way to radioligand therapy, such as in prostate cancer with prostate specific membrane antigen (PSMA) ligand (Sartor et al. 2021) or in neuroendocrine tumours with somatostatin ligand (Strosberg et al. 2017). Among these RPs can be found EMP100, which is a cyclic oligopeptide (Fig. 1) with nanomolar affinity for the human c-MET receptor linked to a DOTA chelator, as measured by fluorescence polarization (3.0 ± 0.5 nM—unpublished results). The oligopeptide was shown to have no pharmacological effect on the HGF/c-Met pathway and was shown not to compete with the native ligand. Gallium-68 radiolabelled EMP100 was investigated in a cohort of 12 metastatic renal cell carcinoma (mRCC) patients with very encouraging results by Mittlmeier et al. (2021). However, in this study the radiolabelling of [^68^Ga]Ga-EMP100 was performed manually. The aim of the present work is to develop an automated method for radiolabelling the EMP-100 peptide with gallium-68 to obtain the RP [^68^Ga]Ga-EMP100.

## Methods

### Chemical precursors, reagents, radionuclide and synthesis module

The EMP100 peptide in lyophilised powder form (30, 50, 75, 90 µg vials) and the [^nat^Ga]Ga-EMP100 standard (500 µg vial) were supplied by Edinburgh Molecular Imaging.

ABX (Advanced Biochemical Compounds, Radeberg, Germany) provided all necessary radiolabel materials in a GMP-compliant single-use kit: 0.08 mol/L ammonium acetate buffer, 60% pure ethanol solution, ascorbic acid, 0.9% NaCl saline, water for injection (WFI, BBraun), eluent solution (5 mol/L NaCl, 0. 1 mol/L HCl), cationic SCX (Bond Elut®, Agilent) and C18 reversed-phase columns (Sep-Pack®, Waters), 0.22 µm filter (Millex-GV®, Merck Millipore LTd.) and sterile vials for the final product, with ultrapure gentisic acid provided by Sigma-Merck.

The gallium-68 eluate was obtained by elution of one or two ^68^Ge/^68^Ga generators Galliapharm® (Eckert and Ziegler GmbH, Germany). The automated synthesis of [^68^Ga]Ga-EMP100 was performed on the GAIA synthesis module (Elysia Raytest, Belgium), placed in a high energy class A laminar air flow hot cell MEDI 5000® (Medisystem, France). This module allows the entire process to be edited and controlled by a computer program.

### Description of the radiolabelling process

**Fig. 2:**
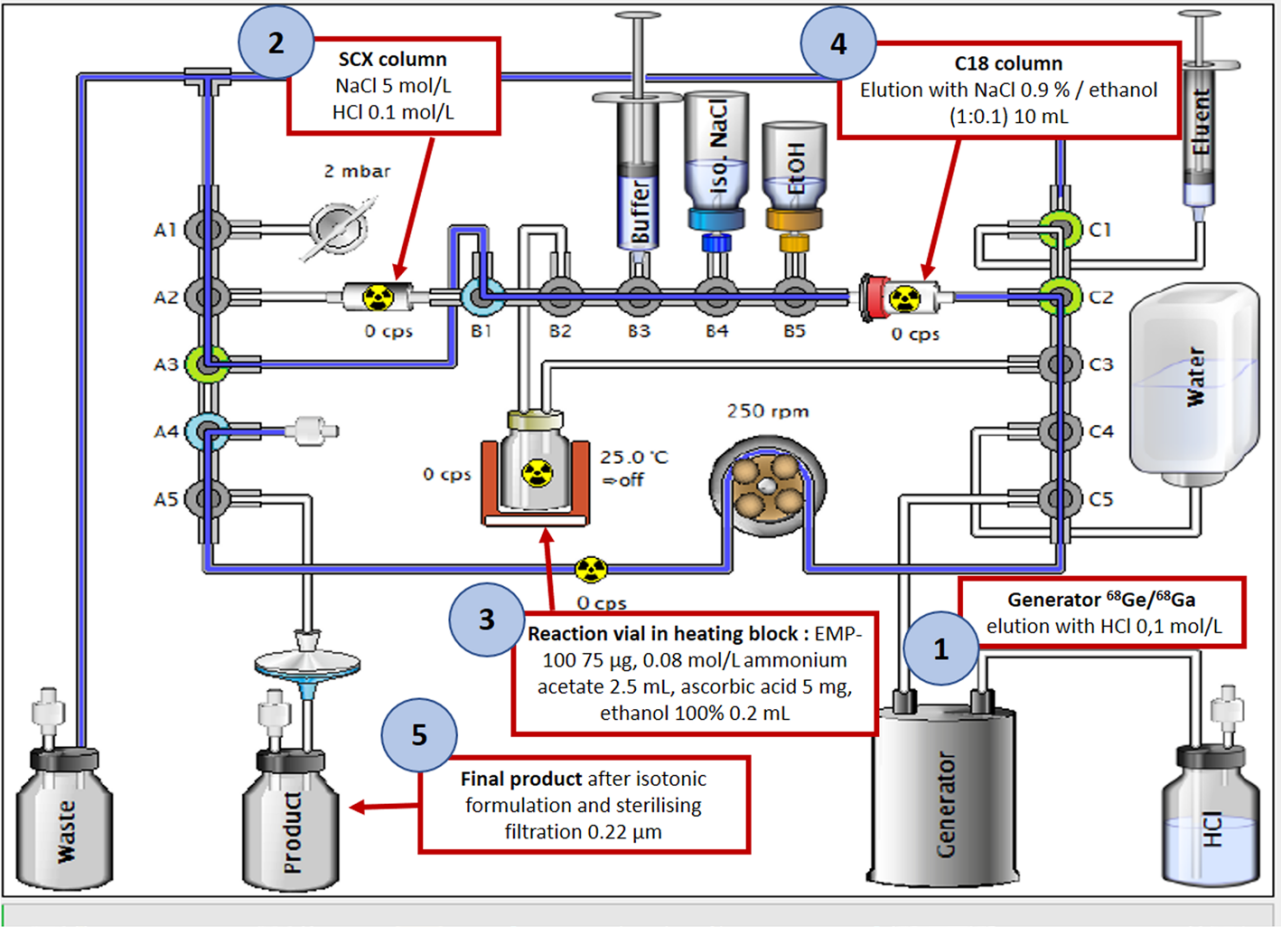
Automated radiolabelling process of [^68^Ga]Ga-EMP100

EMP100 is dissolved with acetate ammonium buffer and then transferred to the reaction vial. The process began with the collection of gallium-68 eluate from the generators on a SCX column, which was then washed from impurities by water for injection (WFI). Gallium-68 was eluted from the SCX column using a 5 mol/L NaCl; 0.1 mol/L HCl solution. The reaction vial containing the EMP100 precursor, gallium-68 ions and adjuvants, was buffered to acidic pH and heated. This was then transferred to a C18 column to trap the [^68^Ga]Ga-EMP100 compound by lipophilic affinity, with the remaining impurities discharged into the waste. The C18 column was then washed with WFI and finally the [^68^Ga]Ga-EMP100 compound was eluted from the column with a mixture of saline and ethanol and then transferred to the final product vial through a 0.22 µm filter. The total radiolabelling time for this fully automated process was approximately 42 min (Fig. 2).

### Optimisation of the radiolabelling process

The three main objectives of radiolabelling optimisation are to improve product quality through a number of specific parameters. Firstly, radiochemical purity (RCP) is controlled and must exceed 95% (EMA. European Medicines Agency 2022, 2018a; Revised guidance for elaborating monographs on radiopharmaceutical preparations: new section on validation of methods 2019). Second, the molar activity (MA) must be greater than 10 GBq/µmol: at this stage of development, this value was chosen based on the current knowledge of needed ratio between ‘hot’ and ‘cold’ compound in the final product as well as commonly achieved values for agents at this stage of development (Bailly et al. 2017; Spreckelmeyer et al. 2020a, b; Jussing et al. 2021). Finally, the target activity at the time of calibration should be greater than 500 MBq, which, combined with a radiochemical yield (RCY) greater than 50%, allows imaging of at least one patient regardless of generator elution yield.

In order for the complexation reaction of Ga^3+^ ions with the oxygen and nitrogen atoms of the DOTA chelate to proceed without problems, the pH must be acidic, preferably buffered between 3.0 and 4.0, with a heat supply (Green and Welch 1989; Kubíček et al. 2010).

To identify the optimal parameters for the complexation of EMP-100 with gallium-68, we used a systematic optimisation approach based on a design of experiments. The optimal parameters for the complexation of EMP-100 with gallium-68 were determined by adjusting the pH (2.75–4.00), temperature (80, 85, 90, 95 °C), heating time (0, 5, 8, 10 and 15 min) with the amount of peptide fixed at 90 µg and without adjuvants. For these first 3 parameters, the RCP > 95% measured by thin layer chromatography (TLC) was the limiting factor for defining the optimised parameter. We performed these experiments in manual mode using the SCX eluate from the module without passing through the C18 column. We then heated the reaction vial using an Elite® heating block (Major Science, Taiwan). In a subsequent step, using the GAIA® module (Elysia Raytest, Belgium) and C18 purification, we optimised the amount of EMP-100 peptide (30, 50, 75, 90 µg) and the effect of adjuvants (ethanol, ascorbic acid and gentisic acid) on the quality of [^68^Ga]Ga-EMP100 in the final product. Optimisation criteria included RCP measured by TLC and HPLC (> 95%), RCY (> 50%) and molar activity (MA) (> 10 GBq/µmol).

The RCP was determined by TLC using a mixture of 1 mol/L ammonium acetate and methanol (1:1), while the RCY was calculated according to the following formula:

$$RCY = \frac{ C18\;activity\;before\;elution - C18\;activity\;after\;elution}{{SCX\;initial\;activity\;measured\;on\;SCX }}*100$$

C18 and SCX activities are automatically recorded during radiolabelling and are available in the final report.

### Validation of quality controls and 3 batches

As the compound [^68^Ga]Ga-EMP100 does not have a monograph in the European Pharmacopoeia (Ph. Eur.), we followed the general text Ph. Eur. 0125 (Radiopharmaceuticals) and Ph.Eur. 51900 (Extemporaneous preparation of radiopharmaceuticals) (Ph. Eur. 11th Edition 2022).

After each radiolabelling, an aliquot of the final [^68^Ga]Ga-EMP100 product was taken for quality control, which included the following parameters.

#### Appearance

Visual inspection of the solution behind a lead glass screen was used to verify that the solution was clear and colorless.

#### Measurement of activity and calculation of molar activity

A MEDI-405® activimeter (Medisystem, France) was used to measure the final radioactivity of the [^68^Ga]Ga-EMP100 product, expressed in MBq, with a lower limit of 500 MBq per 10 mL. The molar activity (MA) was calculated by dividing the radioactivity (GBq) by the amount of precursor peptide (µmol). The lower limit of determination was 10 GBq/µmol.

#### Radionuclide identity

The gallium-68 radionuclide decays by the emission of positrons, whose dematerialisation results in 511 keV gamma photons, which were identified using a Mucha® gamma counter (Elysia Raytest, Belgium). The half-life of the radionuclides in the final product, assumed to be gallium-68 only, was determined by a five-point decay test using a MEDI 405® activimeter. The half-life at each point was measured and calculated according to the monographs Ph. Eur. 0125 and Ph. Eur. 2482, which should be between 61 and 75 min (Ph. Eur. 11th Edition 2022).

#### Radiochemical identity and purity

##### Thin layer chromatography

A 5 µL sample of the final product was applied to a solid iTLC-SG paper stationary phase (Agilent, US) for migration into a mobile phase of 1 M ammonium acetate and methanol (1:1). The compounds were characterised by a retardation factor (Rf), which reflects the migration distance of the compound relative to the spotting line. The [^68^Ga]Ga-EMP100 has an Rf > 0.8, while free gallium-68 impurities and colloidal forms have an Rf < 0.1. The RCP by TLC must be greater than 95%. The TLC papers were analysed using a miniGITA® scanning radiochromatograph (Elysia Raytest, Belgium) and peak integration was performed using GINA® software (Elysia Raytest, Belgium).

##### High performance liquid chromatography (HPLC)

In accordance with ICH Q2 (R1) standards and RP recommendations (Revised guidance for elaborating monographs on radiopharmaceutical preparations: new section on validation of methods 2019; Gillings et al. 2020; Todde et al. 2014; Tietje et al. 2010; EMA. European Medicines Agency 2018b), an HPLC method was developed and validated on a Nexera-i LC 2040C 3D® instrument (Shimadzu, Japan) coupled in series with a diode array detector for UV absorbance detection at 220 nm and 280 nm and a GABI Nova® radioactivity detector (Elysia Raytest, Belgium) for 511 keV photon detection. The reversed phase column used for separation was a Luna Omega 3 µm PS C18® 100 Å, 100 × 4.6 mm (Phenomenex, US). Injections of 5 µL were made at a fixed flow rate of 1 mL/min using a gradient elution mode with solvents A (water/0.1% formic acid), B (acetonitrile/0.1% formic acid) over a period of 12 min. The following phase gradient was applied: 0–1.7 min B 3%, 1.7–8 min B 70%, 8–9 min B 70%, 9–12 min B 3%.

The GINA X® software (Elysia Raytest, Belgium) was used to integrate the different peaks.

The retention time (Rt) of the compound of interest [^68^Ga]Ga-EMP100 was expected to match the retention time of the "cold" standard [^nat^Ga]Ga-EMP100 ± 5%.

Radiochemical purity limits were set for colloidal gallium-68 and free gallium-68 detectable by radio-HPLC and/or TLC.

HPLC was used for chemical and radiochemical identification of the various species likely to be present in the final solution: [^68^Ga]Ga-EMP100, EMP100, degradation or radiolysis products and the free gallium-68 species.

The overall RCP of the compound of interest [^68^Ga]Ga-EMP100 was calculated using the following formula

$$RCP = \frac{{\left( {100 - {\text{A}}} \right) \times {\text{B}}}}{100}$$

where A is the percentage of gallium-68 impurities in free and colloidal form calculated by TLC and B is the percentage of [^68^Ga]Ga-EMP100 determined by HPLC. We have set a target of 95% for the RCP (overall) of [^68^Ga]Ga-EMP100.

#### Radionuclide purity

Each ^68^Ge/^68^Ga Galliapharm® generator was checked on receipt for germanium-68 breakthrough (< 0.001% according to the [^68^Ga]GaCl Ph. Eur. 2464 monograph). The 511 keV peak due to germanium-68 emissions was measured after elution using a Mucha® gamma counter.

#### pH evaluation

For the experiments performed in manual mode, we measured the pH using a Sevencompact duo S213® pH meter (Mettler Toledo) after decay, and for the experiments performed in automated mode, we measured the pH using test strips (VWR and Sigma).

#### Endotoxin, sterility testing and residual solvents

Endotoxin testing or pyrogen evaluation was performed using a chromogenic method on the Endosafe Nexgen® instrument (Charles River, Ireland) with a specification of less than 17.5 IU/mL (Ph. Eur. 11th Edition 2022). Sterility of the finished product was assessed by inoculation into a culture medium after decay (> 48 h) and absence of growth for 14 days, as described (Ph. Eur. 11th Edition 2022).

Ethanol, used to stabilise the complex during the heating step and to elute the product in the purification step, must be less than 10% in the final product (Ph. Eur. 11th Edition 2022). Ethanol is quantified by gas chromatography (GC) (Ph. Eur. 50400).

### Assessment of reproducibility and stability

Three batches of [^68^Ga]Ga-EMP100 were produced to validate the radiopharmaceutical production and quality control process, and each was thoroughly analysed to ensure that all quality parameters met the acceptance criteria. The stability of [^68^Ga]Ga-EMP100 was evaluated over a period of 3 h at room temperature, with RCP measured by TLC and HPLC.

## Results

### Optimisation and validation of [^68^Ga]Ga-EMP100 radiolabelling

The first result of the radiolabelling optimisation was the identification of the optimal pH range between 2.75 and 4.00, which was achieved by varying the volume of 0.08 mol/L ammonium acetate between 1700 and 4400 µL and measuring the effect on the RCP measured by TLC (Table 1), keeping the temperature and heating time at 90 °C for 10 min constant. The highest RCP was obtained with a buffer volume of 2500 µL, corresponding to a pH of 3.75.

**Table 1:** Complexation pH study of [^68^Ga]Ga-EMP100

pH (n = 3)	2.75	3.00	3.25	3.50	3.75	4.00
RCP TLC (%) ± SD	58 ± 0.4	71 ± 12	91 ± 10	90 ± 17	94 ± 10	55 ± 44

Subsequent investigations were carried out on the influence of heating time at a stable temperature (90 °C), measuring the incorporation of gallium-68 by assessing the RCP at different time points (0, 5, 8, 10 and 15 min). A duration of 10 min was found to ensure almost complete incorporation (Table 2).

**Table 2:** Study of the complexation time of [^68^Ga]Ga-EMP100

Heating time (n = 1)	0	5	8	10	15
RCP TLC (%)	67	97	95	99	99

The effect of complexation temperature was then investigated at 80, 85, 90 and 95 °C, with pH and duration maintained at 3.75 and 10 min, respectively. Complexation was found to be almost complete at temperatures of 90 °C and above (Table 3).

**Table 3:** Study of the complexation temperature of [^68^Ga]Ga-EMP100

Temperature (n = 1)	80	85	90	95
RCP TLC (%)	70	96	98	99

Throughout the above optimisation steps, 90 µg of EMP100 peptide precursor was consistently used and RCP by TLC was performed prior to C18 according to the manual mode described above. Smaller amounts of peptide precursor (30, 50, 75 µg) were tested to determine the minimum amount required for satisfactory synthesis yield. Satisfactory RCP (> 95%) and RCY (> 50%) were obtained with 75 µg of precursor peptide (Table 4).

**Table 4:** Summary of the QC data for [^68^Ga]Ga-EMP100 according to the amount of peptide (n = 3 or more for each point)

Amount of EMP100 peptide	30 µg	50 µg	75 µg	90 µg
	8 nmol	13 nmol	20 nmol	24 nmol
RCP TLC (%) ± SD	78 ± 8	89 ± 13	96 ± 3	99 ± 1
RCY n.c.d (%) ± SD	27 ± 7	42 ± 15	57 ± 5	69 ± 5
Molar activity (GBq/µmol) ± SD	28 ± 15	31 ± 8	22 ± 11	23 ± 9

**Fig. 3:**
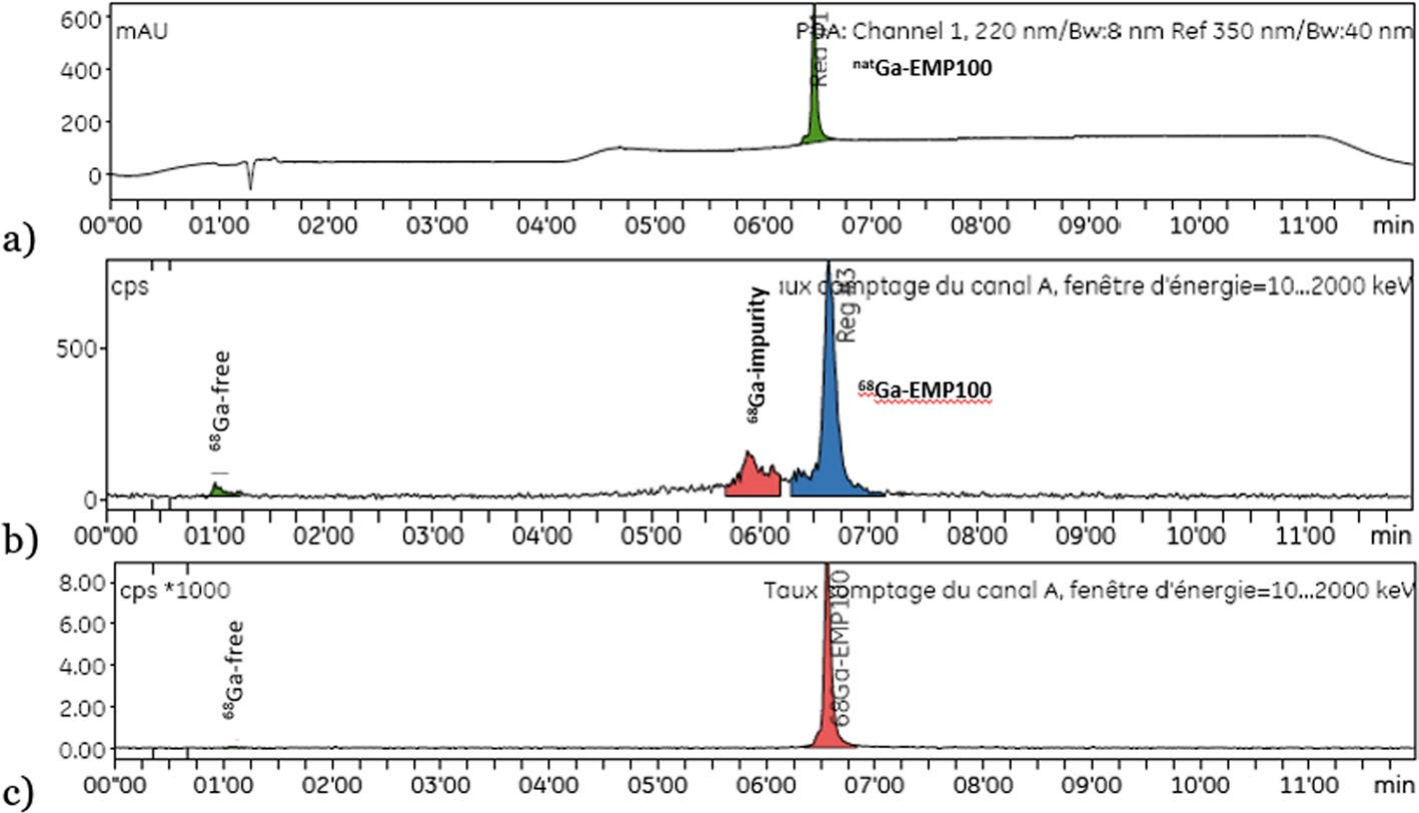
HPLC chromatogram showing: **a** the peak of the cold standard [^nat^Ga]Ga-EMP100 (UV 220 nm), **b** peaks of free [^68^Ga]Ga^3+^ and [^68^Ga]Ga-impurities due to radiolysis before the [^68^Ga]Ga-EMP100 peak after synthesis without adjuvants, **c** the peak of [^68^Ga]Ga-EMP100 after synthesis with adjuvants

When measuring the chemical identity by radio-HPLC, we observed radioactivity peaks that most likely correspond to radiolysis impurities or incomplete radiolabelling (Fig. 3b, Table 5 (Test identification number 1)).

**Table 5:** Summary of [^68^Ga]Ga-EMP100 QC data by adjuvant

Test identification number	1	2	3
n	6	2	13
*Nature of added excipients*			
Ascorbic acid	−	+	+
Ethanol	−	+	+
Gentisic acid	−	+	−
*Main results*			
RCP radio-TLC (%) ± SD	96 ± 3	100	99 ± 1
RCP radio-HPLC (%) ± SD	98 ± 2	100	99.5 ± 0.4
RCY (n.c.d) (%) ± SD	55 ± 9	70 ± 4	68 ± 7

To reduce radiolysis oxidation and stabilise the reaction mixture, we introduced adjuvants such as ascorbic acid, gentisic acid and ethanol prior to the heating step. Initial tests with all three adjuvants gave consistent results as reflected in the RCP and RCY data (Table 5 (column 2), Fig. 3c). Then we tested the combination of ethanol with ascorbic acid on a series of radiolabelling and all batches were within specifications (Table 5 (column 3), Fig. 3c).

Three batches of [^68^Ga]Ga-EMP100 were prepared under the optimised synthesis parameters (pH 3.75, heating temperature 90 °C for 10 min, 75 µg precursor EMP100, with ascorbic acid and ethanol added as adjuvants). All three batches were found to be within the defined specifications (Table 6).

**Table 6:** Results of radiolabelling of [^68^Ga]Ga-EMP100 according to Ph. Eur. 51900

Test	Method	Specifications	Batch 1	Batch 2	Batch 3
Aspect	Visual	Clear, colourless solution	Yes	Yes	Yes
Radioactivity	Activimeter	> 500 MBq	1025 MBq	1065 MBq	1084 MBq
Radioactivity concentration	Activimeter	> 50 MBq/mL	102.5 MBq/mL	106.5 MBq/mL	108.4 MBq/mL
Molar activity	Activimeter	> 10 GBq/µmol	51 GBq/µmol	53 GBq/µmol	54 GBq/µmol
Chemical identification	Radio-HPLC	Rt [^68^Ga]Ga-EMP100 = Rt Standard ± 5%	+ 0.47%	+ 0.62%	+ 0.46%
	Radio-TLC 1M AcNH4 Methanol (1:1)	[^68^Ga]Ga-EMP100: Rf = [0.8;1]	Rf = 0.9	Rf = 1	Rf = 0.9
		[^68^Ga]Ga-colloidal: Rf = [0;0.1]	Rf = 0	Rf = 0	Rf = 0
Radiochemical purity	Radio HPLC	RCP [^68^Ga]Ga-EMP100 > 95.00%	99.64%	99.70%	99.82%
		Free [^68^Ga]GaCl_3_ ≤ 5%	0.36%	0.30%	0.18%
	Radio TLC 1M AcNH4 Methanol (1:1)	[^68^Ga]Ga-EMP100 > 95%	99.44%	99.75%	99.99%
		[^68^Ga]Ga-colloidal ≤ 5%	0.56%	0.25%	0.01%
	RCP (overall)	[^68^Ga]Ga-EMP100 (overall) > 95.00%	99.08%	99.45%	99.81%
pH value	pH-strips	4.0–8.0	6.0	6.0	6.0
Bacterial endotoxins	LAL-test	< 17 EU/mL	< 5 EU/mL	< 5 EU/mL	< 5 EU/mL
Radionuclidic identity	Half-life [^68^Ga]Ga = 67.6 min	Measured:61–75 min	64.92 min	68.23 min	68.13 min
	Gamma spectrometry	0.511 MeV	0.511 MeV	0.511 MeV	0.511 MeV
Radionuclidic purity	Gamma spectrometry	[^68^Ge]Ge < 0.001%	1.12 × 10^–5^	1.08 × 10^–5^	0.95 × 10^–5^
RCY (n.d.c.)	GAIA report	> 50%	75%	72%	75%
Filter integrity test (GAIA)	Membrane filtration bubble point test	YES or NO	Yes	Yes	Yes
Sterility	Inoculation 14 days (Ph Eur 20601)	Sterile	Sterile	Sterile	Sterile
Residual solvents	GC	Ethanol max 10% (v/v)	8.14%	7.74%	6.85%

The stability of [^68^Ga]Ga-EMP100 was assessed over 3 h in the finished product vial at room temperature, measuring appearance, pH, radiochemical purity, and sterility. Results remained within established specifications (Table 7).

**Table 7:** Results of [^68^Ga]Ga-EMP100 stability study in final vial

Test	Method	Specifications	Batch 1	Batch 2	Batch 3
Aspect	Visual	Clear, colourless solution	Yes	Yes	Yes
pH value	pH-strips	4.0–8.0	6.0	6.0	6.0
Stability over 3 h (RCP %)	Radio HPLC	[^68^Ga]Ga-EMP100 > 95,00%	98.63%	98.87%	99.52%
		Free [^68^Ga]GaCl_3_ ≤ 5%	1.37%	1.13%	0.48%
	Radio TLC AcNH4 1 M Methanol (V/V)	[^68^Ga]Ga-EMP100 > 95%	99.85%	99%	99.91%
		[^68^Ga]Ga-colloidal ≤ 5%	0. 15%	1.00%	0.09%
	RCP (overall)	[^68^Ga]Ga-EMP100 (overall) > 95.00%	99.08%	97.87%	99.43%
Sterility	Inoculation 14 days (Ph Eur 20601)	Sterile	Sterile	Sterile	Sterile

## Discussion

Here we describe the pharmaceutical development and validation of an automated method and quality control system for gallium-68 labelling with a c-MET ligand (EMP100) using the Gaia Luna® module.

Gallium-68 is a positron emitter that is readily detectable in PET-CT imaging and has the advantage of being readily available in hospitals thanks to ^68^Ge/^68^Ga generators. As a result, gallium-68 radiopharmaceuticals can be prepared on site without the need for a medical cyclotron. Automated systems are a good solution for gallium-68 radiolabelling because they are more reliable, more reproducible and guarantee consistent yields. As clinical demand increases, process automation also improves operator radiation protection compared to manual methods and meets regulatory requirements.

We have developed a method for radiolabelling EMP100 peptide precursor with gallium-68 using a Gaia Luna® module (Elysia Raytest) to obtain [^68^Ga]Ga-EMP100. This method has many advantages. Firstly, by trapping the gallium-68 eluate on a cationic column prior to the radiolabelling process, the radioactivity can be concentrated, allowing pooling of generator elution to achieve higher activities. Another advantage of cationic eluate purification is the ability to remove concentrations of zinc ions (from gallium-68 decrease) and other metallic impurities that could compete with gallium-68 labelling reactions. The cationic SCX method also allows control of the volume of the reaction mixture, ensuring constant pH and reproducible complexation of gallium ions by the chelator (Velikyan 2015; Mueller et al. 2012; Zhernosekov et al. 2007; Meisenheimer et al. 2020; Nelson et al. 2022).

To achieve successful radiolabelling of EMP100, we optimised the critical production parameters such as pH, heating time, complexation temperature and amount of peptide. The optimum reaction pH was found to be 3.75 using ammonium acetate buffer. The pH of the buffer plays an important role in radiolabelling procedures, particularly with gallium-68, and the reaction kinetics for ^68^Ga^3+^ incorporation is inversely related to pH (Bartholomä et al. 2010; Bnzeth et al. 1994). We observed the best radiolabelling efficiency at pH 3.75 and note that at pH 4, hydrolysis to insoluble ^68^Ga(OH)_3_ occurs in the preparation, as the radiolabelling process is inconsistent at low to normal RCP. This range of pH is in agreement with previously published results for the manual radiolabelling of [^68^Ga]Ga-EMP100, carried out by fractionated elution of gallium-68, where the pH used was between 3.7 and 4.0, obtained using sodium acetate (Mittlmeier et al. 2021). The reaction temperature was then investigated: this is an important factor since, above a certain temperature, gallium-68 ions can form both gallium oxides and hydroxides as precipitates (Silva et al. 2009), and some biological compounds, such as peptides, can be thermolabile and undergo degradation or denaturation, thus affecting the quality of the final RP (Lepareur 2022). While published data show radiolabelling at 95 °C for 15 min, our investigations show that incorporation is complete after 10 min and that a heating temperature of 90 °C is sufficient for complete complexation.

Molar activity (GBq/µmol) is an important parameter in PET imaging. When the biological target concentration is minimal, image quality and quantification can be improved by a high MA, as has been shown for GLP-R (Velikyan 2015; Velikyan et al. 2017, 2008; Migliari et al. 2022; Eriksson et al. 2014) for insulinoma imaging using ligands such as exendin-4. In particular, the presence of unlabelled peptide can reduce the concentration of radioactivity in the target tissue due to competition with the labeled peptide for the same receptor. In a manual process, Mittlmeier et al. (2021) used 100 µg of EMP100 precursor, corresponding to 27 nmol. In this study, we investigated different amounts to find the minimum required and found that above 75 µg of peptide (equivalent to 20 nmol), a sufficient synthesis yield is achieved, i.e. above 50%, with an RCP in line with specifications.

During radiolabelling of [^68^Ga]Ga-EMP100, gallium-68 atoms decay, emitting gamma and beta radiation. In the presence of water molecules in the solution, this radiation generates oxygenated free radicals. These radical species are capable of oxidising certain biological molecules, particularly thiol groups and certain amino acids (methionine, cysteine, isoleucine) (Velikyan 2015; Meisenheimer et al. 2020; Velikyan et al. 2017; Janota et al. 2016). During the initial measurement of chemical identity by radio-HPLC, we observed gallium-68 peaks, probably corresponding to radiolysis impurities, at earlier retention times compared to the main [^68^Ga]Ga-EMP100 peak. To reduce the oxidative effect of radiolysis and to further stabilise the reaction medium, we introduced various adjuvants known to have antioxidant effects and found consistent results in terms of RCP, RCY and radiochemical identification when the combination of the two excipients (ascorbic acid and ethanol) were added.

Finally, three consecutive batches of [^68^Ga]Ga-EMP-100 were produced according to the parameters defined during optimisation and were found to meet the defined specifications. In addition, the product was found to be stable 3 h after radiolabelling. Based on a starting activity of 1500 MBq, automated radiolabelling yielded approximately 1000 MBq of final product with a final MA of 50 GBq/µmol. This would be sufficient to image 2 or 3 (70 kg) patients at 2.0 MBq/kg body weight with a single PET camera, as with the gallium-68-labelled radiopharmaceuticals [^68^Ga]Ga-PSMA-11 (EMA. European Medicines Agency 2022; Fourquet et al. 2021) and [^68^Ga]Ga-DOTATOC (EMA. European Medicines Agency 2018a; Delabie et al. 2022; Moreau et al. 2022) already used in clinical routine.

This robust automated radiolabelling process helps to achieve the highest possible MA, i.e. with the smallest amount of peptide that allows sufficient gallium-68 incorporation yield. In clinical practice, this starting MA allows injection of [^68^Ga]Ga-EMP100 even after a decay time of up to 2 h, although the MA is lower than the specification (10 GBq/µmol).

## Conclusion

For the automated radiolabelling of [^68^Ga]Ga-EMP100, the parameters of pH, temperature, precursor peptide content, and the use of adjuvants for impurity management were efficiently optimised, resulting in the production of 3 compliant and stable batches according to the principles of good manufacturing practice. [^68^Ga]Ga-EMP100 was successfully synthesised and is now available for clinical development in PET-CT imaging.

## Data Availability

All data generated and analysed during this study are included in this published article.

## Abbreviations

c-MET: Tyrosine-protein kinase Met
DOTA: Tetraxetan or 2,2′,2′′,2′′′-(1,4,7,10-Tetraazacyclododecane-1,4,7,10-tetrayl)tetraacetic acid
d.c.: Decay corrected
EMI: Edinbourg Molecular Imaging
FISH: Fluorescent in situ hybridization
HGF: Hepatocyte growth factor
HPLC: High-performance liquid chromatography
ICH: International Council for Harmonisation of Technical Requirements for Pharmaceuticals for Human Use
IHC: Immunohistochemistry
GC: Gas chromatography
GLP-R: Glucagon-like peptide-1 receptor
GMP: Good manufacturing practice
LAL: Limulus amebocyte lysate test
MA: Molar activity
n.d.c.: Non-decay corrected
NGS: Next-generation sequencing
NSCLC: Non-small cell lung cancer
TLC: Thin layer chromatography
Ph. Eur.: European Pharmacopoeia
PSMA: Prostate specific membrane antigen
PET-CT: Positron emission tomography coupled with computed tomography
QC: Quality control
RCP: Radiochemical purity
RCY: Radiochemical yield
Rf: Rate of flow
RP: Radiopharmaceutical drug
Rt: Retention time
SCX: Solid cationic exchange
SD: Standard deviation
SST-R: Somatostatin receptors
SUV: Standardized uptake value
